# Postpartum spontaneous vulvar hematoma as a cause of maternal near miss: a case report and review of the literature

**DOI:** 10.1186/s13256-022-03281-2

**Published:** 2022-02-28

**Authors:** Temesgen Tilahun, Aaga Wakgari, Aschalew Legesse, Rut Oljira

**Affiliations:** 1 Department of Obstetrics and Gynecology, Institute of Health Sciences, Wollega University, Oromia, P.O Box: 395, Nekemte, Ethiopia; 2 Department of Public Health, Institute of Health Sciences, Wollega University, Nekemte, Ethiopia

**Keywords:** Vulvar hematoma, Postpartum, Severe anemia, Maternal near miss

## Abstract

**Background:**

Postpartum spontaneous vulvar hematoma is a rare complication of childbirth that can potentially cause maternal death if not managed properly and in a timely manner.

**Case summary:**

We present the case of maternal near miss secondary to postpartum hemorrhage secondary to vulvar hematoma after home delivery in a 28-year-old para IV mother from rural Ethiopia. The case was surgically managed under spinal analgesia. The mother and her newborn were discharged on the fourth postprocedure day.

**Conclusion:**

Neglected and inappropriately managed postpartum vulvar hematoma can cause significant maternal morbidity; therefore, timely surgical exploration, ligation of bleeding vessels, and obliteration of dead space can avert severe maternal complications

## Introduction

According to the World Health Organization (WHO), maternal near miss (MNM) is defined as “a woman who nearly died but survived a complication that occurred during pregnancy, childbirth or within 42 days of termination of pregnancy” [1]. In Ethiopia, for every woman who dies from pregnancy-related causes, 12–21 others experience maternal near miss (MNM) [1–3]. One of the causes of MNM is postpartum hemorrhage (PPH) [1, 4, 5]. The major causes of PPH are uterine atony, genital tract laceration, retained tissues (placenta and membranes), and coagulopathy [4, 5]. Vulvar hematoma is among genital tract traumas that cause PPH [5, 6].

A vulvar hematoma is a collection of blood in the vulva [6]. The vulva is soft tissue mainly composed of smooth muscle and loose connective tissue and is supplied by branches of the pudendal artery [7, 8]. The venous drainage is provided by labial veins, and labial veins drain into the external and internal pudendal veins [8, 9]. This vulvar vasculature commonly develops varicosities during pregnancy, especially in parous women, due to increased venous pressure created by the increasing weight of the uterus [8]. Damage to labial branches of the internal pudendal artery in this vascular network easily initiates hematoma development [7–9].

Postpartum vulvar hematomas cause maternal morbidities such as anemia, postpartum hemorrhage, superinfection, necrotizing fasciitis, prolonged hospitalization, and need for transfusion [5–7]. Here we present, a case of postpartum vulvar hematoma as the cause of MNM.

## Case presentation

This is a 28-year-old para IV mother from rural Ethiopia who gave birth to an alive female neonate weighing 3000 g at home 24 hours before presentation. She did not remember her last normal menstrual period (LNMP) but claimed to be amenorrheic for 9 months. She had antenatal care (ANC) at a nearby health center where she had routine investigations and care during pregnancy. During the last antenatal visit, she was told to come to the health center when she feels labor pains. However, her labor advanced within 6 hours of the onset of labor pain. She gave birth normally at home with the assistance of traditional birth attendants. The mother reported that there was neither difficulty with delivery of the placenta nor excessive bleeding during and after delivery. She noticed gradual swelling of her right vulva that was associated with vulvar pain. Due to the worsening of these complaints, the family took her to nearby hospital. At this hospital, evacuation of vulvar hematoma (6 cm × 8 cm) was done. The managing team evacuated 500 ml of hematoma and referred the patient to Wollega University Referral Hospital (WURH) for blood transfusion.

Upon arrival to WURH, the patient was re-evaluated by the charge resident physician and consultant gynecologist, and obstetrician. The mother reported vaginal bleeding from the vulva on her way to WURH, significant swelling of the vulva, and vulvar pain. She had difficulty with micturition. She also complained of palpitation, easy fatigability, vertigo, and headache. The patient had no history of hypertension, diabetes mellitus, or bleeding tendency.

**Fig. 1:**
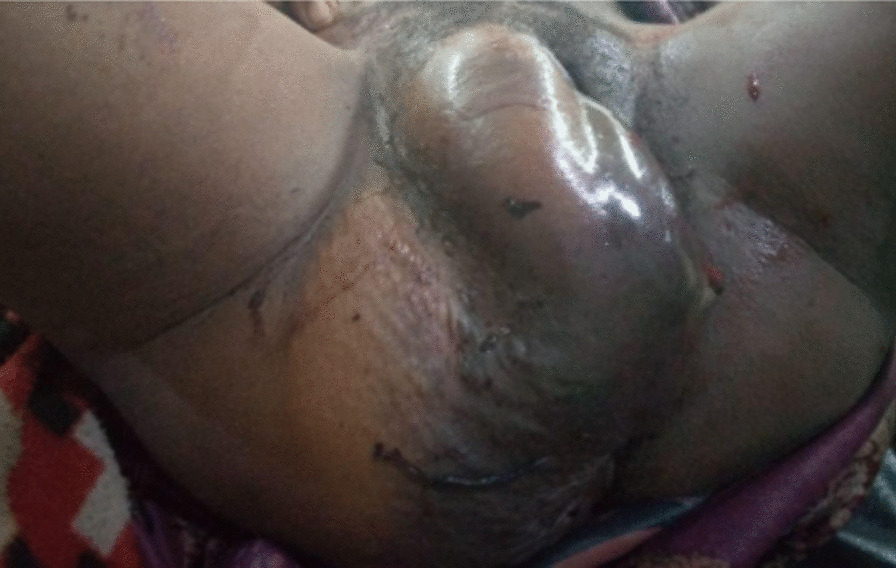
Postpartum spontaneous vulvar hematoma managed at Wollega University Referral Hospital, Western Ethiopia, 2021

On examination, she was acutely sick-looking. Her vital signs were blood pressure (BP) 90/60 mmHg, pulse rate (PR) 136 beats per minute, respiratory rate (RR) 22 breaths per minute, and temperature 37.1 °C. She had dry buccal mucosa and pale conjunctivae. Lymph glandular system, chest, and cardiovascular system were normal. Abdominal examination showed a 20-week-sized uterus that was well contracted and nontender, and the bladder was distended. There were no signs of fluid collection or organomegaly. On genital examination, there was a 12 × 20 cm right-sided vulvar mass extending to the mons pubis and posteriorly to the right buttock (Fig. 1). The mass was tender and fluctuant. There were two stitches applied to it. However, there was bleeding from this site. There was no active vaginal bleeding or vaginal and cervical tear. She exhibited palmar pallor. On neurologic examination, she was oriented to time, person, and place. She had normal reflexes and no neurologic deficits. With the final diagnosis of severe anemia secondary to postpartum hemorrhage secondary to a vulvar hematoma, she was admitted to the obstetric ward. At admission, she was investigated and the results were as indicated in Table 1.

**Table 1:** Summary of laboratory investigations of the case of postpartum spontaneous vulvar hematoma managed at Wollega University Referral Hospital, Western Ethiopia, 2021

Time of investigations	Laboratory tests	Results
At admission	CBC count	WBC count 1760 cells/μl; RBC count 1.1 million cells/μL; hematocrit 10.3%^a^; platelet count 189,000 cells/μl; MCV 92.2 fL; MCH 30.6 picograms(pg)
	Urinalysis	Nonrevealing
	RBG	145 mg/dl
	Blood group	B+
	Abdominopelvic ultrasound	Empty uterus, no peritoneal collection
	VDRL	Nonreactive
	HBsAg	Nonreactive
After procedure	CBC count	WBC count 1971 cells/μl; RBC count 1.4 million cells/μl; platelet count 177,000 cells/μl; MCV 92.1 fL; MCH 30.7 picograms(pg)
		Hematocrit 12.9%^b^
		Hematocrit 17.2%^c^

*CBC* complete blood count, *WBC* white blood cell, *RBC* red blood cell, *VDRL* Venereal Disease Research Laboratory, *HBsAg* hepatitis B surface antigen, *RBG* random blood glucose, *MCV* mean corpuscular volume, *MCH* mean corpuscular hemoglobin

^a^At admission

^b^After the first transfusion

^c^After the second transfusion

The patient was prepared and taken to the operation room. Under spinal analgesia, through a previous incision made at referring hospital, about 700 ml of clotted blood was evacuated from vulvar hematoma. The actively bleeding vessels were identified and ligated. Then, the wound was sutured in three layers. The site was observed for bleeding and vulvar swelling. A hemostatic gauze was used for further compression and removed after 12 hours. The patient was transferred to ward where she was transfused with two units of compatible blood. On the fourth postprocedure day, the patient was discharged with ferrous sulfate and appropriate advice on vulvar care.

## Discussion

This is the case of maternal near miss in rural Ethiopia. The major causes of maternal near-miss events are obstetric hemorrhages, hypertensive disorders of pregnancy, difficult labor and delivery, sepsis, complications of abortion, and uterine rupture [1, 5]. This patient presented with postpartum hemorrhage (PPH) secondary to spontaneous vulvar hematoma. It is an unusual cause of PPH [5, 6]. The other causes of PPH such as uterine atony retained tissue, coagulopathy, and genital tract laceration were excluded from patient history, physical examination, and laboratory investigation. A huge postpartum vulvar hematoma explained the patient’s condition. The hematoma was severe enough to cause maternal shock and severe anemia. This patient could have died had she not been aggressively managed with intravenous fluid, blood transfusion, and surgical intervention.

Postpartum vulvar hematomas are rare events in modern obstetrics. Their magnitude varies from 1 per 300 to 1 per 15,000 deliveries. It can be classified into obstetric and non-obstetric vulvar hematomas [6, 8, 10, 11]. Postpartum vulvar hematomas most frequently result from genital tract laceration [8] or improper hemostasis during the repair of perineal tears or an episiotomy wound. Failure to take precautions while suturing the apex of the episiotomy may result in a large vulvovaginal hematoma due to the distensible nature of the tissue [12, 13].

Postpartum spontaneous vulvar hematomas are rare events. They result from injury to blood vessels in the absence of laceration or incision of the surrounding tissue (such as pseudoaneurysm and traumatic arteriovenous fistula) [10, 13]. They usually follow precipitate labor, macrosomic babies, prolonged second stage of labor, hypertensive disorders of pregnancy, coagulopathy, or vulvar varicosities [2, 10, 11, 14]. In our case, the total duration of labor was only 6 hours, which might have been the triggering factor. It occurred spontaneously after home vaginal delivery. It is observed that most spontaneous vulvar hematomas are right-sided vulvar hematomas [11] as in our case. This may be due to dextrorotation of the uterus, which might cause vulvar varicosities.

The pathogenesis of vulvar hematomas is due to iatrogenic injury to blood vessels and/or spontaneous rupture resulting in various symptoms such as vulvar swelling, vulvar pain, and urologic symptoms [6, 8, 10, 15]. Our patient presented with vulvar swelling, vulvar pain, and difficulty with urination. As bleeding into the vulva is largely restricted only by the Colles fascia and the urogenital diaphragm, a hematoma in this area is visible as tender fluctuant mass [15] as in our case.

Vulvar hematomas may develop within hours after delivery or be initially misdiagnosed as vulva swelling or edema until the delayed formation of the hematoma [8, 14]. Early recognition is paramount in reducing the associated morbidity, improving patient outcomes, and shortening the length of hospital stay. Delay in recognition and management may result in adverse consequences and increase maternal morbidity [7, 11, 14] as in our case.

The management of vulvar hematomas depends on the size of the hematoma, hemodynamic stability of the patient, availability of medical resources, and duration of the hematoma [9, 10, 14, 16]. Smaller and chronic vulvar hematomas can be conservatively managed [9] including the use of sitz baths, ice packs, empiric antibiotics, pain medication, and/or blood transfusion [12, 14]. However, large and rapidly expanding hematomas, as in this case, are managed by surgical techniques [9, 11]. The surgical management can be surgical exploration or selective arterial embolization [9, 10]. The surgical exploration consists of incision and drainage of the hematoma, ligation of the bleeding vessels, and packing or placement of drainage tube [9–11]. Our patient was managed by a similar approach. However, the primary treating hospital did not place a vaginal pack or drainage tube. As a result, the patient was having ongoing active bleeding from the incision site on the way to the referral hospital. This made the patient develop a recurrent huge hematoma. Therefore, optimal management of vulvar hematomas includes surgical exploration, ligation of bleeding vessels, obliteration of the dead space and placing pack in the vagina, placing drainage tube, or applying pressure over it [9, 10, 14]. The surgical exploration also prevents pressure necrosis of the surrounding tissue and decreases the risk of infection and necrotizing fasciitis [6, 8]. Sometimes, however, a surgical repair may fail or a recurrent hematoma can be formed, as in our case. In such cases, selective arterial embolization is the treatment of choice [10, 17].

## Conclusion

Neglected and inappropriately managed postpartum vulvar hematoma can cause significant maternal morbidity; therefore, timely surgical exploration, ligation of bleeding vessels, and obliteration of dead space can avert maternal complications

## Data Availability

The datasets used during the current study are available from the corresponding author on reasonable request.

## Abbreviations

ANC: Antenatal care
CBC: Complete blood count
HBsAg: Hepatitis B surface antigen
LNMP: Last normal menstrual period
MCH: Mean corpuscular hemoglobin
MCV: Mean corpuscular volume
MNM: Maternal near miss
PPH: Postpartum hemorrhage
RBC: Red blood cell
RBG: Random blood glucose
VDRL: Venereal Disease Research Laboratory
WBC: White blood cell
WUR: Wollega University Referral Hospital
